# Molecular Dissection of Cyclosporin A’s Neuroprotective Effect Reveals Potential Therapeutics for Ischemic Brain Injury

**DOI:** 10.3390/brainsci3031325

**Published:** 2013-09-05

**Authors:** Minoru Kawakami

**Affiliations:** Aquatic Animal Health Division, National Research Institute of Aquaculture, Fisheries Research Agency, Tamaki, Mie 519-0423, Japan; E-Mail: minkawa@gmail.com; Tel./Fax: +81-96-372-8602

**Keywords:** ischemic cell death, neuroprotective agent, cyclosporin-A, transcriptional regulation

## Abstract

After the onset of brain ischemia, a series of events leads ultimately to the death of neurons. Many molecules can be pharmacologically targeted to protect neurons during these events, which include glutamate release, glutamate receptor activation, excitotoxicity, Ca^2+^ influx into cells, mitochondrial dysfunction, activation of intracellular enzymes, free radical production, nitric oxide production, and inflammation. There have been a number of attempts to develop neuroprotectants for brain ischemia, but many of these attempts have failed. It was reported that cyclosporin A (CsA) dramatically ameliorates neuronal cell damage during ischemia. Some researchers consider ischemic cell death as a unique process that is distinct from both apoptosis and necrosis, and suggested that mitochondrial dysfunction and Δψ collapse are key steps for ischemic cell death. It was also suggested that CsA has a unique neuroprotective effect that is related to mitochondrial dysfunction. Here, I will exhibit examples of neuroprotectants that are now being developed or in clinical trials, and will discuss previous researches about the mechanism underlying the unique CsA action. I will then introduce the results of our cDNA subtraction experiment with or without CsA administration in the rat brain, along with our hypothesis about the mechanism underlying CsA’s effect on transcriptional regulation.

## 1. Introduction

Brain ischemic stroke is a common neurological disease and a leading cause of severe disability and death in Western countries. It is proposed that the extent of tissue injury observed after ischemia largely reflects the damage incurred during reperfusion [1]. So, if we have effective methods for controlling ischemic reperfusion injury, we expect good outcomes in our therapeutic approach to stroke within a substantial time window following the onset of stroke.

After the onset of brain ischemia, a series of events leads ultimately to the death of neurons [2,3]. Many molecules can be pharmacologically targeted to protect neurons [4] during these events, which include glutamate release, glutamate receptor activation, excitotoxicity, Ca^2+^ influx into cells, pH changes, mitochondrial dysfunction, adenosine triphosphate depletion, activation of many intracellular enzymes, free radical production, nitric oxide production, apoptosis, and inflammation [2].

In this review, I discuss the current problematic situation in the development of an effective neuroprotectant for ischemia and focus on CsA as an example of a promising candidate neuroprotectant. Then, I review our own work on CsA.

Currently, there are very few successful neuroprotectants. Edaravone (MCI-186) is one of a few effective neuroprotective agents that have already reached clinical application, although it is used only in a limited number of countries [5,6]. It acts as an antioxidant and a strong free radical scavenger, protecting against oxidative stress and neuronal apoptosis [7,8,9]. There have been a number of other attempts to develop effective neuroprotective agents for brain ischemia. However, many of these have not succeeded. In the next section, I will exhibit some examples of representative neuroprotectants that have been attempted or that have advanced to clinical trials (Table 1). 

## 2. Neuroprotective Drugs

The following are examples of agents that were tested or are under clinical trial (Table 1).

### 2.1. Antioxidants

#### 2.1.1. NXY-059 (Cerovive)

A 2005 phase III clinical trial [10,11] demonstrated some efficacy in the acute treatment of ischemic injury due to stroke. However, a 2006 attempt to repeat this trial in a larger number of cases indicated no significant activity. The development program was then terminated.

**Table 1:** Neuroprotective drug candidates and details of their clinical trials.

Categories	Drugs	Current status/Result of clinical trial
Antioxidants	NXY-059 (Cerovive)	Trial in a larger number of cases indicated no significant activity. The program was terminated.
	Tirilazad	The drug increased the combined endpoint of “death or disability” by about one-fifth.
	Nicaraven	The drug did not demonstrate enough therapeutic efficacy in the treatment of acute ischemic stroke.
	Ebselen	A phase III trial exploring the efficacy in patients with cortical infarct is under way.
	SUN-N8075	A phase I trial is under way in the United States.
Calcium Antagonists	Nimodipine	A clinical trial once suggested a beneficial effect, but none of the subsequent trials confirmed this result.
	Monosialoganglioside GM1	There is not enough evidence to conclude that gangliosides are beneficial in acute stroke.
	Nootropil (Piracetam)	There is not enough evidence to assess the effect of piracetam in acute ischemic stroke.
	E-2051	A phase I clinical trial is under way in Europe.
N-methyl-D-aspartate (NMDA) Receptor Antagonists	Selfotel	The drug was not effective and might have a neurotoxic effect in brain ischemia.
	Gavestinel	There is no evidence of benefit. Further development has been discontinued.
	Magnesium	No beneficial effects on the functional outcome of stroke in patients were accrued except in cases of lacunar syndromes.
GABA Agonists	Clomethiazole	The drug did not improve outcome in patients with major ischemic stroke.
Sodium Channel Blockers	lubeluzole	The drug failed to show the efficacy in the treatment of acute stroke.
Opioid Antagonists	nalmefene	A phase III study was completed, but the results were not published.
Membrane Stabilizers	Citicoline	The drug was not efficacious in the treatment of moderate-to-severe acute ischemic stroke.
	DP-b99	The Phase III trial is under way.
Poly ADP-ribose Polymerase (PARP) Inhibitors	ONO-2231	A Phase I clinical trial is under way in the UK.
	AX200 (Granulocyte Colony-Stimulating Factor (G-CSF))	The drug failed to show the efficacy in the treatment of acute stroke.
Immunosuppressants	FK506 (Tacrolimus)	A human stroke trial was stopped in phase II.
	Cyclosporin A	A Phase II clinical trial, Neuroprotection Impact of Cyclosporin A in Cerebral Infarction (CsAStroke), is under way.
PTP inhibitors	Pramipexole(Mirapex)	No information about clinical trial for an inhibitor of PTP.
	S-15176	No information about clinical trial for an inhibitor of PTP.
Others	Albumin	A Phase III randomized multicenter clinical trial of high-dose human albumin therapy for neuroprotection in acute ischemic stroke.
	ONO-2506 (Proglia)	A Phase III crinical trial in acute stroke patients was completed and failed to show the efficacy of the drug in the treatment of acute stroke.
	SUN-N4057 (Piclozotan)	Phase II clinical trials are under way in the USA and Europe.
	TS-011	Phase I clinical trials of TS-011 are under way.

#### 2.1.2. Tirilazad

When administered to patients with acute ischemic stroke, Tirilazad mesylate increased the combined endpoint of “death or disability” by about one-fifth but did not alter case fatality [12].

#### 2.1.3. Nicaraven

Nicaraven did not demonstrate enough therapeutic efficacy in the treatment of acute ischemic stroke, and its clinical trial application was declined [13,14].

#### 2.1.4. Ebselen

Patients diagnosed as having acute ischemic stroke and who could receive drug treatment within 48 h of stroke onset were enrolled in one study. The improvement was significant in patients who started ebselen within 24 h of stroke onset but not in those who started treatment after 24 h. A phase III trial exploring the efficacy of ebselen in patients with cortical infarct is under way [15,16,17].

#### 2.1.5. SUN-N8075

SUN-N8075 is a radical scavenger with neuroprotective properties. It also exerts blockade effects against T-type calcium channels as well as sodium channels. A phase I trial is under way in the United States [18].

### 2.2. Calcium Antagonists

#### 2.2.1. Nimodipine

Its efficacy in reducing infarct volume and improving outcome was demonstrated in animal models of focal ischemia, but subsequent clinical trials in stroke patients gave conflicting results. A clinical trial once suggested a beneficial effect [19], but none of the many subsequent trials confirmed these results [20,21].

#### 2.2.2. Monosialoganglioside GM1

There is not enough evidence to conclude that gangliosides are beneficial in acute stroke [22].

#### 2.2.3. Nootropil (Piracetam)

There is not enough evidence to assess the effect of piracetam in acute ischemic stroke [23].

#### 2.2.4. E-2051

In rat cortical slices and synaptosomes, E-2051 inhibited increases in glutamate and intracellular Ca^2+^, respectively, elicited by high K^+^, in a concentration-dependent manner. A phase I clinical trial is under way in Europe [24].

### 2.3. *N*-Methyl-D-Aspartate (NMDA) Receptor Antagonists

#### 2.3.1. Selfotel

Selfotel was not an effective treatment for acute ischemic stroke. Furthermore, a trend toward increased mortality, particularly within the first 30 days and in patients with severe stroke, suggested that the drug might have a neurotoxic effect in brain ischemia [25]. The development of this drug has been discontinued.

#### 2.3.2. Gavestinel

There is no evidence of benefit from excitatory amino acid antagonists for acute stroke [26]. Further development of this drug has been discontinued [27,28].

#### 2.3.3. Magnesium

Magnesium inhibited NMDA receptors as well as voltage-dependent calcium channels (VDCCs). Magnesium is neuroprotective in animal models of stroke, and findings of small clinical pilot trials suggest a potential benefit to people. However, the present study [29] failed to demonstrate statistically significant stroke recovery even though serum magnesium levels rose significantly, indeed more than that necessary for neuroprotection by an intravenous magnesium sulfate regime. Therefore, in spite of the beneficial effects of magnesium therapy on the histological and functional outcomes of cerebral ischemia in animal models, no beneficial effects on the functional outcome of stroke in patients were accrued except in cases of lacunar syndromes. Further clinical trials are necessary to determine the benefits, dose, duration, and timing of magnesium therapy to reduce morbidity and mortality in lacunar syndromes [30].

### 2.4.GABA Agonists: Clomethiazole

In clinical trials, Clomethiazole did not improve outcome in patients with major ischemic stroke [31,32].

### 2.5.Sodium Channel Blockers: Lubeluzole

Studies failed to show the efficacy of lubeluzole in the treatment of acute stroke. Further development of this product has been stopped [33,34,35,36].

### 2.6.Opioid Antagonists: Nalmefene

Although nalmefene appears to be safe and well tolerated, a study failed to find any treatment benefit in stroke patients treated within 6 h after onset. A phase III study was completed, but the results were not published. There is currently no active development of this drug for neuroprotection [37].

### 2.7. Membrane Stabilizers

#### 2.7.1. Citicoline

Citicoline (cytidine-5-diphosphocholine; CDP-choline) is a naturally occurring nucleotide derivative that may reduce central nervous system (CNS) ischemic injury by stabilizing cell membranes and reducing free radical generation [38]. The drug once seemed to show some evidence of efficacy in a pooled analysis. However, citicoline did not improve recovery at 90 days after moderate-to-severe acute ischemic stroke. Under the circumstances of that trial (International Citicoline Trial on acUte Stroke (ICTUS)), citicoline was not efficacious in the treatment of moderate-to-severe acute ischemic stroke [39,40,41,42].

#### 2.7.2. DP-b99

DP-b99 is a lipophilic moderate-affinity chelator of zinc and calcium ions that acts selectively within cell membranes and has neuroprotective properties in animal models of stroke [43]. The Phase III, Membrane-Activated Chelator Stroke Intervention trial is under way. It is based on promising data derived from previous Phase I and II DP-b99 trials and capitalizes on lessons learned from failures of past stroke studies in relation to neuroprotection, patient selection, and data analysis [44].

### 2.8. Poly ADP-Ribose Polymerase (PARP) Inhibitors

#### 2.8.1. ONO-2231

ONO-2231, a PARP inhibitor, is under development for the treatment of acute phase cerebral ischemic stroke. As PARP is an enzyme involved in cellular death, ONO-2231 is expected to be effective for diseases such as acute stroke. A Phase I clinical trial of ONO-2231 is under way in the UK [24].

#### 2.8.2. AX200 (Granulocyte Colony-Stimulating Factor (G-CSF))

G-CSF stimulates the Signal Transducers and Activators of Transcription 3 (STAT3) signaling pathway and inhibits PARP. The drug, AX200, is a manufactured form of G-CSF. AX200 stops neuronal cell death in the acute phase of stroke and stimulates the regeneration of the already damaged nervous tissue through the stimulation of neurogenesis as well as arteriogenesis and the reorganization of neuronal networks. It demonstrated safety and efficacy in patients with acute stroke in phase IIa clinical trials in Europe which have been completed. Further development is planned [45].

According to the recent report, they were not able to show efficacy for AX200 as a treatment for acute ischemic stroke. These results are disappointing and were unexpected to them because AX200 showed signs of efficacy in a previous clinical trial with a limited number of patients, as well as in numerous animal studies [46].

### 2.9. Albumin

Exogenous human serum albumin administration has been found to be neuroprotective via reducing brain swelling, prevention of post-ischemic thrombosis, anti-oxidant activity, hemodilution and increasing the perfusion to the ischemic tissue [47]. Preliminary results from the Albumin in Acute Stroke (ALIAS) Part 1 suggest a trend toward a favorable primary outcome in subjects treated with 25% human albumin (ALB) and support the validity of the statistical assumptions that underlie the ALIAS Part 2 Trial. The ALIAS Part 2 Trial is under way and will confirm or refute these results [48,49,50,51].

### 2.10. ONO-2506 (Proglia)

Arundic acid (AA; ONO-2506), a novel modulator of astrocyte activation, significantly increased the mRNA expression of glutamate transporters (GLT-1 and GLAST) and GABA receptors (GABAA-R β1, GABAA-R β2, and GABAA-R β3). At a similar concentration range, the agent significantly decreased the mRNA expression of S-100β and NGF-β, as well as the lipopolysaccharide-stimulated mRNA expression of inducible nitric oxide synthase (iNOS) and cyclooxygenase-2 (COX-2) [52]. A Phase III trial in acute stroke patients was completed [45] and failed to show the efficacy of ONO-2506 in the treatment of acute stroke [53].

### 2.11. SUN-N4057 (Piclozotan)

SUN-N4057 (Piclozotan) is a novel benzoxazepine derivative that has selective affinities to the 5-HT1A receptors. SUN-N4057 displayed pronounced neuroprotective activity compared with conventional neuroprotective agents, such as glutamate antagonists and antioxidants, in a rat model of middle cerebral artery occlusion (MCAO) [54]. In addition, SUN-N4057 showed efficacy even when administered 3–6 h after the onset of cerebral ischemia in rats and gerbils. Furthermore, SUN-N4057 exerted marked neuroprotective activity in a cat model of focal cerebral ischemia. In pharmacological safety studies, no serious effects on the CNS or cardiovascular system were noted. The results of these preclinical studies indicate that SUN-N4057 is a potent neuroprotective agent with a good safety profile compared with other neuroprotective agents. Phase I clinical studies were completed using healthy male volunteers in the UK, and phase II clinical trials are under way in the USA and Europe [45].

### 2.12. TS-011

Cytochrome P450s metabolize arachidonic acid to hydroxyeicosatetraenoic acids (HETEs) and epoxyeicosatrienoic acids (EETs). Among these, 20-hydroxyeicosatetraenoic acid (20-HETE) plays an important role in the regulation of vascular tone in the brain [55]. TS-011 is a 20-HETE-synthesizing enzyme inhibitor; it reverses vasospasm in stroke and suppresses ischemic neuronal death. Phase I clinical trials of TS-011 are under way. 

## 3. Immunosuppressants

### 3.1. FK506 (Tacrolimus)

In T-cells, activation of the T-cell receptor normally increases intracellular calcium, which acts via calmodulin (CaM) to activate calcineurin. Calcineurin is a serine/threonine phosphatase that is regulated by Ca^2+^/CaM and that dephosphorylates the transcription factor nuclear factor of activated T-cells (NF-AT), which moves to the nucleus of the T-cell and activates the transcription of gene coding for interleukin-2 (IL-2) and related cytokines. FK506 prevents the dephosphorylation of NF-AT [56]. In detail, FK506 reduces peptidyl-prolyl isomerase activity by binding to the immunophilin FKBP12 (FK506 binding protein), creating a new complex. This FKBP12-FK506 complex interacts with and inhibits calcineurin, thus inhibiting both T-lymphocyte signal transduction and IL-2 transcription [57].

In 1994, Sharkey reported for the first time that FK506 was neuroprotective following cerebral ischemia [58], and many animal experimental studies have examined this effect [59,60,61]. It was revealed that FK506 exhibits both neuroprotective and neuroregenerative properties through various mechanisms, including the ability to cross the blood brain barrier (BBB) [62,63], inhibitory action against cytochrome c release, anti-inflammatory action [64], and suppression of the death protein BAD [65,66]. Another study in an ischemic model found that FK506 administration was associated with a significant downregulation of interleukin-1beta (IL-1beta) expression in astrocytes and microglia, suggesting that these are targets for FK506 and that the modulation of glial response and inflammation may be a mechanism of FK506-mediated neuroprotection in ischemia [67]. FK506 was also shown to protect markedly against demyelination and axonal loss in a model of multiple sclerosis (MS) through immunosuppression and neuroprotection [68]. Its use has also been explored in traumatic brain injury (TBI) and spinal cord injury (SCI) [45]. On the other hand, however, a human stroke trial was stopped in phase II [1].

### 3.2. Cyclosporin A

Cyclosporin A (CsA) is a cyclic nonribosomal peptide of 11 amino acids produced by the fungus *Tolypocladium inflatum*, and is a widely used strong immunosuppressant. CsA binds to the cytosolic protein cyclophilin in T-cells, and the CsA-cyclophilin complex inhibits calcineurin [57,69].

It was reported that CsA dramatically ameliorates neuronal cell damage in the CA1 sector of the hippocampus during forebrain ischemia [70,71,72]. CsA inhibits the Ca^2+^-induced mitochondrial membrane permeability transition (MPT) but might be unrelated to a reduction in post-traumatic Ca^2+^ accumulation [73]. MPT is a calcium-triggered increase of mitochondrial membrane permeability that leads to loss of Δψ, mitochondrial swelling, and rupture of the outer mitochondrial membrane, and is caused by the opening of a channel known as permeability transition pore (PTP). PTP consists of the voltage-dependent anion channel (VDAC), the adenine nucleotide translocator (ANT), cyclophilin-D (CypD: a mitochondrial peptidyl prolyl-*cis*, *trans*-isomerase), and other molecule(s) [74]. MPT can form only if it has CypD available. CsA binds up all CypD within each mitochondrion. CsA-treated mitochondria continue to function normally, even while under attack from conditions damaging the brain.

At least in animal models, CsA was reported to be effective in the treatment of TBI. For example, post-injury treatment with CsA significantly reduced mitochondrial dysfunction marker alpha-spectrin degradation in a mouse model [75]. CsA was capable of blunting *N*-acetylaspartate (NAA) reduction and restoring ATP loss, two sensitive markers of mitochondrial dysfunction, in a rat model [76]. CsA treatment also resulted in a significant reduction in amyloid precursor protein (APP) mRNA, and neuronal perikaryal APP antigen expression in a large animal model using sheep [77]. Also, in animal experiments, intravenous CsA achieves therapeutic levels in brain parenchyma, and 10 mg/kg is the most effective dose in attenuating axonal damage after TBI [78]. The NIH has funded multicenter human trials for treating TBI with CsA. Potential applications are as follows:

- Acute: TBI, SCI, stroke, and cerebral vasospasm following subarachnoid hemorrhage (SAH).
- Chronic: neurodegenerative diseases such as amyotrophic lateral sclerosis (ALS), Huntington’s disease (HD), Parkinson’s disease (PD), and Alzheimer’s disease (AD).
- Selective neuronal brain protection from radiotherapy for brain cancer.

Based on these good outcomes of animal experiments, a prospective, randomized, placebo-controlled Phase I/II trial was performed and published in 2009 with the aim to evaluate the safety, tolerability, and pharmacokinetics of a single intravenous infusion of CsA in patients with severe TBI [79]. Fifty adult patients with severe TBI were enrolled over a 22-month period and received treatment within 12 h of the injury. Although CsA was not associated with significant adverse events compared to placebo, there was no significant difference in neurological outcomes. Also in a prospective, blinded, placebo-controlled, randomized, dose-escalation trial of intravenous CsA initiated within 8 h of TBI, the rate of mortality or other adverse events was not significantly different from that in the placebo group [45,80].

On the other hand, a clinical study of 50 adults with severe TBI demonstrated the beneficial effect of 24 h of CsA treatment on brain extracellular metabolites using *in vivo* microdialysis. Glutamate concentration and lactate/pyruvate ratio were significantly higher in the placebo group than in CsA treated patients, respectively one to two days, and two to three days after the end of the 24-h drug infusion. The administration of CsA was also associated with a significant increase in mean arterial pressure (MAP) and cerebral perfusion pressure (CPP) [81].

A Phase II clinical trial, Neuroprotection Impact of Cyclosporin A in Cerebral Infarction (CsAStroke), is under way. The main objective of the study is to determine whether or not a single injection of CsA after intravenous thrombolysis can significantly decrease the volume of cerebral infarction [82]. Secondary objectives are to determine whether a single injection of CsA after intravenous thrombolysis is safe and effective regarding to death and disability. The study started in October 2009 and was expected to be completed in December 2012. No study results have been published so far.

## 4. Agents That Modulates/Inhibits PTP

### 4.1. CsA as a Neuroprotective Agent

Some researchers consider ischemic cell death as a unique molecular process that is distinct from both apoptosis and necrosis, and the accumulated evidence suggests that mitochondrial dysfunction and Δψ collapse are key steps in ischemic cell death [83].

As described above, CsA has a unique neuroprotective effect that is considered to be closely related to the molecular mechanisms of ischemic cell death in neurons *per se* [74]. There are two previously reported target molecules in neurons: calcineurin and CypD.

The well-known calcineurin function is a target for the widely used immunosuppressive molecules CsA and FK506 [57], as described above. Immunophilins (cyclophilins and FKBP12s) the binding proteins of CsA and FK506, respectively play important roles in the inhibition of calcineurin and in the immunosuppressive effect. It should be noted that calcineurin is extremely enriched in neural tissue [84]. In neurons, calcineurin can act as a Ca^2+^-buffering protein [85], and another report suggests that calcineurin exerts neuroprotective effects by increasing the expression of the antioxidant superoxide dismutase (SOD), via nuclear factor (NF) κB after cerebral ischemia [86]. It was demonstrated that an interaction between the antiapoptotic Bcl-2-family and calcineurin activity was important in the regulation of cell death during apoptosis [87], and that calcineurin specifically participates in a Ca^2+^-inducible mechanism for apoptosis induction by regulating the phosphorylation of Bad, a Bcl-2 proapoptotic family member [65,88].

CypD, on the other hand, is an important modulator of the MPT pore, as described above. MPT is a Ca^2^^+^-dependent increase of mitochondrial membrane permeability that leads to the loss of Δψ, mitochondrial swelling, and rupture of the outer mitochondrial membrane. CypD is essential for MPT to occur, and CypD-dependent MPT regulates some forms of necrotic, but not apoptotic, cell death. The anti-apoptotic proteins Bcl-2 and Bcl-xL can block MPT and can therefore block MPT-dependent necrosis in addition to their well-established ability to inhibit apoptosis [74].

Calcineurin and CypD are distinct and separate key pharmacological targets of neuroprotective agents. Many previous reports have attributed the neuroprotective effects of CsA to pathways related to either or both of those target molecules. Calcineurin alone may not explain CsA’s neuroprotective effect, because it was suggested that FK506’s neuroprotective effect is much weaker than that of CsA in ischemia, despite the fact that the inhibitory effect of FK506 on calcineurin is 10 times stronger than that of CsA [89]. The CypD model also cannot account for all of the protective effects. A knockout study of CypD [90] reported the following results.

(1) CypD deficiency resulted in a significant reduction of hypoxic-ischemic brain injury in adult mice but worsened injury in neonates.
(2) CypD-deficient cells also responded to various apoptotic stimuli in a manner similar to that of the WT, suggesting that CypD is not a central component of the apoptotic death pathway.

Taken together, these data suggested that unidentified events underlie the CsA-mediated protective effect.

### 4.2. Other PTP Inhibitors

#### 4.2.1. Pramipexole (Mirapex)

Pramipexole, an aminobenzothiazole compound, is a dopamine agonist with high spcificity for the D2 dopamine receptor family. Activation of the postsynaptic D2 receptor subtype provides the most robust symptomatic improvement in PD. Given its pharmacological profile, it is not surprising that pramipexole was found to be effective in ameliorating parkinsonian signs in animal models [91].

Several reports suggested that the PTP is a possible target of this drug [92,93,94]. Pramipexole has been shown to enter and accumulate in mitochondria driven by the mitochondrial membrane potential [93]. Targeting to mitochondria has also been recently comfirmed by patch clamp experiments showing inhibition of PTP by pramipexole [94]. PTP inhibition by pramipexole was further supported by data obtained in functional intact mitochondria showing that this drug prevented Ca^2+^-triggered swelling of mitochondria [94,95].

Although Pramipexole (Mirapex) has been widely used as a dopamine agonist, there is no information about its clinical application as a PTP inhibitor.

#### 4.2.2. S-15176

S-15176, *N*-[(3,5-di-tertiobutyl-4-hydroxy-1-thiophenyl)]-3-propyl-*N*′-(2,3,4-trimethoxybenzyl) piperazine is a partial fatty acid oxidation inhibitor, which induces a shift from fatty acid oxidation to glucose oxidation in heart and liver metabolism, finally leads to a reduced gluconeogenesis and improved energy metabolisms of these organs. This inhibitory effect contributes to the anti-ischemic effects of the drug [96,97].

Apart from a partial fatty acid oxidation inhibition, S-15176 was reported to prevent PTP opening. This effect was closely related with an increase in the Ca^2+^ loading capacity of mitochondria. Although S-15176 was a strong inhibitor of lipid peroxidation, experiments with another trimetazidine derivative lacking antioxidant activity demonstrated that this activity was not essential to the inhibitory effect. Binding studies elucidated mitochondrial binding sites of S-15176, especially those localized in the inner membrane, which were shared by several well-known inhibitors of PTP opening. These results strongly suggest that the mechanism by which S-15176 protects mitochondria against the deleterious effects of ischemia-reperfusion involves inhibition of PTP opening and provide evidence that the drug exerts its effect through low structural specificity binding sites located in the inner mitochondrial membrane [98]. So far, there is no information about a clinical trial of S-15176 as a PTP inhibitor.

## 5. CsA’s Effect on Transcriptional Regulation

As described above, CsA’s effect on T-cell activation is based on effect on the transcription of gene coding for IL-2. Also, it was reported that CsA affects the transcription of brain-derived neurotrophic factor (BDNF) in neurons [99]. So, it is possible that some genes play important roles in ischemic cell death and are transcriptionally regulated by unknown specific pathways that CsA affects. To test this possibility, our group performed a cDNA subtraction experiment between CsA-treated brain and control brain [100]. Next, I describe our cDNA subtraction experiment.

### 5.1. Analysis of the Effects of CsA Administration on Gene Expression

The rat brain ischemia model has been widely used as a standard by which to estimate a drug’s effect. We used Wistar rats as model animals. For the cDNA subtraction method, we used the suppression-subtractive hybridization (SSH) method. SSH is an advanced PCR-based cDNA subtraction method with high sensitivity [101]. This method has the following advantages over the Gene-chip method:

- easy and cost-effective
- applicable when levels of starting material are minimal
- does not require prior knowledge of the transcriptomes
- can detect not only mRNA but also a variety of other RNA species (e.g., miRNA, siRNA)
- can obtain candidate cDNA clones during the analysis.

However, it also has disadvantages compared to Gene-chip:

- Gene-chip is fast, simple, and shows more coverage
- Gene-chip does not need cloning or sequence steps.

### 5.2. Materials and Methods

#### 5.2.1. CsA Administration

CsA was injected into the rat brains as previously described [72,89]. Male Wistar rats weighing 270–330 g, corresponding to ~9–10 weeks of age, were used in the experiments (*n* = 12 per group). A needle was inserted into each side of the hippocampus. Using a 10 mL Hamilton syringe, 1 mL of saline solution was deposited stereotactically into each side of the hippocampus at two cannula penetrations. CsA was infused at a dose of 10 mg kg^−1^ (Novartis, Täby, Sweden) into the carotid arteries. After 6 h, the brains were perfused with physiological saline for 1 min just before decapitation. For control animals, the same amount of vehicle was used, using the same protocol.

#### 5.2.2. Tissue Preparation and RNA Preparation

The animals were sacrificed under anaesthesia and the brains were removed and dissected. The hippocampal CA1 region was isolated and quick-frozen in liquid nitrogen. Total RNA was extracted from frozen tissues obtained from multiple animals, and Poly-A+ RNA was isolated from the total RNA.

#### 5.2.3. cDNA Subtraction and Isolation of the Candidate Clones

This method is described in detail by Diatchenko *et al*. [101]. Subtractions were made in both forward and reverse directions (Forward: CsA-control, Reverse: Control-CsA). A total of 187 clones from the forward pool and 252 clones from the reverse pool of the cDNA were randomly selected and cloned into the vector. After plasmid purification, an identical amount of each isolated plasmid DNA was blotted onto two nylon membranes. For screening, each of the duplicated membranes was hybridized by a labeled probe created from the cDNA mixture synthesized from the forward pool or the reverse pool. To confirm the results, each membrane was hybridized by two probes: one created from control rat brain cDNA and the other created by CsA-treated rat brain cDNA. After hybridization, signals were detected by autoradiography. cDNA clones that showed differences in a pair of signal strengths on duplicated membranes were selected, sequenced, and analyzed. Chimeric clones were excluded from analysis.

### 5.3. Results of cDNA Subtraction Experiment

We detected many genes that were upregulated or downregulated by CsA administration. Among those, nine significantly upregulated genes and seven significantly downregulated genes were detected following CsA administration [100].

#### 5.3.1. Significantly Upregulated Genes

Elongation factor-1 gamma (EF-1-gamma): EF-1-gamma is one sub-unit of a multi sub-unit protein complex EF-1. EF-1 is a cellular protein complex that plays a role in protein synthesis by mediating the transfer of aminoacyl-tRNA to 80 S ribosomes [102]. Expression of the Ef-1-alpha gene decreases with age in mice and human fibroblasts [103] and forced expression of EF-1-alpha prolongs the lifespan of Drosophila melanogaster [104]. These reports suggest that the EF-1 complex has trophic effects on cellular function. Three sub-units of the EF-1 complex (EF-1-beta, gamma and delta) were shown to bind to DNA modified with chromium and transplatin, which are known to be potent DNA damaging agents [105]. One report showed that Ef-1-delta mRNA levels are increased in cells following exposure to ionizing radiation [106]. These results suggest the possibility that the EF-1 complex may have a damage-response function.

Stearoyl-CoA desaturase 2 (SCD2): SCD2 catalyses a key step in the biosynthesis of oleic acid, a major fatty acid in myelin [107,108]. Remyelination is an essential step during recovery from certain kinds of brain damage [109].

Beta sub-unit of chaperonin-containing TCP1: Chaperonin-containing TCP1 (CCT) is a heterooligomeric molecular chaperone that mediates folding of cytosolic proteins such as tubulin or actin in eukaryotes [110,111]. CCT is also involved in vesicular trafficking [112] and neurite development in neuronal cells [113].

Fasciculation and elongation protein zeta1 (FEZ1): FEZ1 is a mammalian homologue of nematode gene unc-76. UNC-76 was shown to be necessary for normal axonal bundling and elongation within axon bundles in the nematode *C. elegans* [114]. FEZ1 is also identified as a protein kinase C zeta (PKC zeta)-interacting protein and is reported to be located downstream of PKC zeta and to stimulate neuronal differentiation of PC12 cells [115].

14-3-3 eta: 14-3-3 family member proteins are known to regulate PKC activity, exocytosis and the cell cycle [116] and have anti-apoptotic effects [117,118]. Many reports have shown that caspase inhibitors reduce neuronal death after brain ischemia [119,120,121,122,123]. Over-expression of anti-apoptotic genes also reduced ischemic neuronal cell death [124,125]. 14-3-3 eta is known to block apoptosis by inhibiting the activation of p38 MAPK [126].

Syndecan-3: Syndecan-3 is known to be a direct binding partner of the peptide Y-P30, which crosses the blood-placenta barrier and accumulates in foetal cerebral neurons. There, it enhances survival of thalamic neurons and displays neuritogenic activities [127]. In adult rats, Y-P30 expression is also reported to be induced after optic nerve crush [128].

Ataxin-7-like protein 3: Ataxin-7-like protein 3 is reported to be a component of the TATA binding protein (TBP)-free-TBP-associated factor (TAF) complex/suppressor of yeast transposable elements (SPT) 3-TAF9-general control non-derepressible (GCN) 5 acetyltransferase (TFTC/STAGA) complex [129], that has been shown to play a role in ligand-dependent gene activation by nuclear receptors. The TFTC/STAGA complex is evolutionarily well-conserved and its wide range of biological functions renders it indispensable for normal cell function [130]. A polyglutamine expansion in Ataxin-7 has been identified as the mutation responsible for the inherited neurodegenerative disorder, spinocerebellar ataxia type 7 [129].

G-protein-coupled receptor 37 (Gpr37): Northern blot analysis revealed that Gpr37 is expressed in the central nervous system (CNS) [131] and in the testes [132]. In 2006, Rezgaoui *et al*. [133] reported that head activator (HA) is a high-affinity ligand for GPR37. The peptide HA was originally isolated and characterized from hydra, where it mediates head-specific growth and differentiation processes, hence, its name. HA was isolated with an identical sequence from mammalian brain and intestine [134]. HA is present during early mammalian development and is expressed in cells of the nervous system and neuroendocrine system. In adult mammals, HA enhances neurite outgrowth and is neuroprotective. As in hydra, mammalian HA stimulates entry into mitosis and proliferation of cell lines.

ZW10 interactor (ZWINT): ZWINT is thought to be a component of the kinetochore [135]. ZWINT has been implicated in the regulation of faithful chromosomal segregation through binding to the kinetochore [136]. Also, it was reported that ZWINT, RAB3C and synaptosomal-associated protein 25 (SNAP-25) extensively co-localize in synapses of primary hippocampal neurons, suggesting a new role for the kinetochore protein ZWINT in pre-synaptic events that are regulated by RAB3 and SNAP25 [137].

#### 5.3.2. Significantly Downregulated Genes (Group 1)

Significantly downregulated genes were classified into two groups with respect to their functions. The first group encodes proteins that are reported to be detrimental to neuronal cells.

Complement protein C1q beta: C1q is the first component of the classical complement pathway (CCP). *In vitro*, C1q binds to fibrillar beta amyloid peptide (Abeta) and activates the CCP [138,139]. In the Alzheimer’s disease (AD) brain, C1q has been shown to be associated with fibrillar Abeta plaques and activated glial cells [140]. Previously published evidence suggests that CCP activation occurs *in vivo* and can initiate an inflammatory reaction through the recruitment of activated glia [141,142] that could lead to enhanced neurodegeneration [143,144]. It could also facilitate the rapid removal of damaged cells in the initial stages of CNS injury. Thus, C1q, because of its diverse functional capabilities, could play a dual role in the neuropathology [145]. Increasing evidence indicates that CCP activation contributes to ischemia-reperfusion injury in different organs [146,147]. Ten *et al*. [148,149] have reported that neonatal mice with genetic deletion of C1q are protected against hypoxic-ischemic brain injury.

Rab6b: Accumulation of aggregation-prone proteins activates protein quality control mechanisms, in order to prevent toxic activity. An important site for protein quality control is the endoplasmic reticulum (ER). RAB6 is a small GTPase that is involved in vesicular transport and mediates a coat protein 1 (COP1)-independent, retrograde trafficking route from the Golgi to the ER [150,151]. RAB6B is predominantly expressed in brain tissue. RAB6 has been linked to AD in previous studies; dominant negative RAB6 inhibits the formation of Abeta [152]. Scheper *et al*. [153] suggested that initially RAB6 is protective, but that it may become destructive when activation is prolonged.

Visinin-like Ca^2+^ binding protein-1 (VILIP-1): VILIP-1 is a member of a superfamily of neuronal calcium sensor proteins [154]. VILIP-1 expression enhances the neurotoxic effect of ionomycin at low ionophore concentrations, suggesting that VILIP-1 has a role in calcium mediated neurotoxicity [155]. VILIP-1 was found to be associated with fibrillar tangles in AD brains and VILIP-1 expression in PC12 cells enhanced hyperphosphorylation of tau protein [155]. These results suggest that the calcium sensor protein may be involved in the pathophysiology of AD.

#### 5.3.3. Significantly Downregulated Genes (Group 2)

These genes were reported to be important for oxidative metabolism.

Cytosolic malate dehydrogenase (cytMDH): Malate dehydrogenase (MDH) catalyses the NAD/NADH-dependent inter-conversion of malate and oxaloacetate in the cytoplasm (cytMDH) and mitochondria (MitMDH). cytMDH is a part of the malate-aspartate shuttle, controlling the brain mitochondrial NADH/NAD^+^ balance. Impairments in this shuttle may enforce anaerobic metabolism [156].

Oxidation resistance 1 homologue: The oxidation resistance 1 (Oxr1) gene was discovered in a search for human genes that function to protect against oxidative damage [157]. In the C57BL/6 J mouse retina, the expression of the early response protein OXR1 is biphasic during the 14-day period of hyperoxia examined. OXR1 is upregulated at three days, when photoreceptors are resistant to hyperoxia and are downregulated by 14 days, when the photoreceptors have begun to die [158]. It may be better that Oxr1 is downregulated at the early stages of ischemia, since the environment is rather hypoxic.

Adenylate kinase 3 (AK3): AK3 is present in the mitochondrial matrix and functions in transferring the high-energy phosphate to AMP from GTP synthesized by the TCA cycle. The ADP thus generated can then be phosphorylated to ATP by oxidative phosphorylation. Therefore, AK3 function may be a cellular coping mechanism for energy demand in the brain. It is reported that AK3 protein increases ~10-fold during neural differentiation [159]. Furthermore, the AK3 mRNA level in HeLa cells increases in response to hypoxia and this increase depends on the presence of hypoxiainducible factor-1 (HIF-1), a heterodimeric basic helix-loop-helix protein of the Per/Arnt/Sim (PAS) family [160]. Eight potential binding sites for HIF-1 are found in the bovine AK3 promoter [161].

X-linked phosphoglycerate kinase 1: Phosphoglycerate kinase 1 (PGK-1) is the enzyme required in the sixth step of the mammalian glycolytic pathway. It is reported that PGK-1 is upregulated by low oxygen tension. It is reported that the lack of functional HIF-1 complex completely abrogates the response of PGK-1 to hypoxia. Increasing evidence shows that this enzyme is a multifunctional molecule. PGK-1 can affect DNA replication and repair in the mammalian nucleus [162] and stimulate viral mRNA synthesis in the cytosol [163]. This enzyme may also act as an mRNA binding protein and be involved in post-transcriptional regulation of urokinase-type plasminogen activator receptor mRNA [164]. Moreover, PGK-1 may play a role in inhibiting tumour angiogenesis by promoting the extracellular formation of angiostatin from plasmin [165].

### 5.4. Discussion

Our study demonstrates the following points:

1. CsA appears to affect the expression of many genes related to neuronal cell survival and regeneration.
2. Many of the significantly upregulated genes identified in the present study are reported to be neurotrophic or have roles in the regeneration of damaged neurons.
3. CsA reduces the expression of some genes that were reported to be detrimental to neuronal cells.
4. CsA reduces the expression of certain genes that appear to be important for oxidative metabolism.

All of the significantly upregulated genes identified in our study are reported to be neurotrophic or to have roles in the regeneration of damaged neurons. EF-1-gamma, Syndecan-3, and Ataxin-7-like protein 3 are reported to have trophic effects on cells. SCD2 helps to generate the myelin sheath. CCT is a molecular chaperone and is reported to be involved in vesicular trafficking and neurite development. FEZ1 is known to stimulate neural differentiation of PC12 cells. The 14-3-3 family of proteins has an anti-apoptotic effect. GPR37 plays significant roles in brain development and human disease. Also, ZWINT is involved in faithful chromosomal segregation and pre-synaptic events in hippocampal neurons. In contrast, although three significantly downregulated genes (complement protein C1q beta, RAB6B, and VILIP-1) are known to facilitate the cell death pathway, expression of the other four genes is not reported to be detrimental to neuronal cells. cytMDH, OXR1, AK3, and PGK-1 are all related to oxidative metabolism.

Oxidative metabolism regulation is very important to neuronal survival, especially in ischemic conditions or during the process of ischemia-reperfusion injury. At least under normal conditions, cytMDH, AK3, and PGK-1 are thought to be important for energy production in the brain. In addition, the transcription of two genes (AK3 and PGK-1) is reported to be positively regulated by hypoxia-inducible factor-1 (HIF-1). However, it is possible that the expression of these genes increases ischemia-reperfusion injury by producing oxidative stress. On the other hand, OXR1 is known not only to be induced by hypoxia but also to have an important function in protecting against oxidative damage [157]. The relationship between the decrease in OXR1 and CsA’s neuroprotective effects is unclear.

There is a report that immunosuppressive drugs are neurotrophic [63], a finding that is consistent with our data except for the case of OXR1. FK506 is known to have nerve-regenerative properties [166]. Our data suggest that CsA may also have some effect to promote nerve regeneration by regulating the expression of several genes. Also, there are close relationships between AD and the functions of three genes (complement protein C1q beta, RAB6B, and VILIP-1), the expression levels of which are reduced by CsA administration. This is consistent with a previous report that CsA has a neuroprotective effect in AD. Furthermore, CsA is reported to be applicable not only to AD but also to other chronic neurodegenerative diseases such as ALS, HD, and PD, as described above. Taken together these results suggested that, in these cases, CsA exerts neuroprotection via some mechanisms other than the inhibition of MPT pore opening, because these diseases are not necessarily accompanied by mitochondrial dysfunction. Also, some reports show that FK506 has a neuroprotective effect even though FK506 does not interact with CypD [167]. Calcineurin is the mutual target of both CsA and FK506, and the calcineurin NF-AT pathway is proven to affect transcriptional regulation in T-cells and other cells. So, it is likely that CsA affects the expression of genes via the calcineurin-dependent pathway. However, we have no evidence so far that the calcineurin pathway truly affects the expression levels of genes that we detected in the cDNA subtraction. On the other hand, many of the previous reports characterized the MPT pore modulator CypD as a target molecule of CsA in neuroprotection. A knockout study of CypD [90] indicated that CypD deficiency resulted in a significant reduction of hypoxic-ischemic brain injury in adult mice, and CsA did not show a protective effect in this strain. These findings suggest that the CypD/permeability transition pore (PTP) is critical for the development of brain injury and for the effect of CsA. However, no reports have suggested that CypD affects transcription. The separation of the calcineurin-dependent and -independent effects of cyclosporin must be greatly helped by non-immunosuppressive analogs of CsA (such as MeVal-4-Cs, NIM811, and Debio 025) that maintain the ability to bind to and inhibit cyclophilins but not calcineurin [168,169,170,171,172].

**Figure 1:**
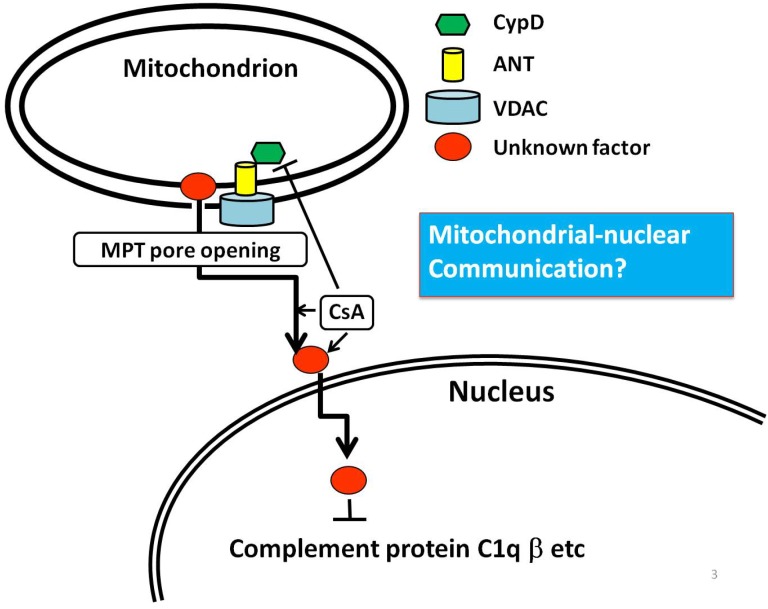
A model of signaling pathways includes hypothetic transcriptional factor (red ellipse) downstream from CypD (green hexagon). Mitochondria have a special protein pore that is formed in the membranes under certain pathological conditions, such as traumatic brain injury and stroke. This pore is the mitochondrial membrane permeability transition pore, or MPT pore. CypD is a modulator of this pore. VDAC (voltage-dependent anion channel) (blue cylinder) and ANT (adenosine nucleotide translocase) (yellow cylinder) are main components that constitute the MPT pore. An unknown factor resides in mitochondria and detects the MPT pore opening regulated by CypD. This factor then translocates from mitochondria to the nucleus and affects transcriptional regulation.

One possible mechanism that could solve this inconsistency is that an unknown factor resides in mitochondria and detects the MPT pore opening regulated by CypD, and then this factor translocates from the mitochondria to the nucleus and affects transcriptional regulation. A knockout study found that CypD deficiency worsened hypoxic-ischemic brain injury in neonates [90]. Our results for cDNA subtraction revealed that CsA administration decreased the expression level of complement protein C1q beta, and a previous report showed that neonatal mice with C1q deletion were protected against hypoxic-ischemic brain injury [148,149]. So, it is consistent that this factor suppresses the transcription of detrimental molecules including complement protein C1q beta in the nucleus after the translocation from mitochondria, assuming that CsA assists in this process via its interaction with CypD (Figure 1). In the next section, I will introduce an example of a factor that translocates from mitochondria to the nucleus.

## 6. An Example of Mitochondria-Nuclear Communication; Prohibitin 1 (PHB1)

PHB1 is a 32 kDa protein that belongs to a family of proteins that share an evolutionarily conserved stomatin/prohibitin/flotillin/HflK/C (SPFH) domain. It is closely related to PHB2, also called B-cell receptor-associated protein 37 (BAP 37) and repressor of estrogen receptor activity (REA). The prohibitin amino acid sequence is highly conserved across species [173,174]. The best-characterized function of the prohibitins is as chaperones involved in the stabilization of mitochondrial proteins [175]. At the subcellular level, PHB1 has been localized to the cell membrane and mitochondrial inner membrane complexed to PHB2 [175,176], as well as the cytoplasm, or the nucleus depending on the cell type and situation [177,178]. Recently, it was demonstrated that PHB1 is translocalized from mitochondria to the nucleus as a mitochondrial response to oxidative stress *in vitro* [179]. This finding is consistent with the implication of PHB1 in diverse cellular processes, including the regulation of proliferation, apoptosis, and gene transcription [173,177,178,180]. PHB1 is an ideal candidate hypothetic factor, as described above. We are planning to test the hypothesis that PHB1 links the transcriptional effect of CsA with mitochondrial status.

## 7. Conclusion

Brain protection therapy is urgently required. In pre-clinical studies, over 1000 potential neuroprotective therapies have been trialed targeting some of the molecular events described above, with many of these treatments providing protection [181]. However, after nearly 200 clinical trials, almost all attempts at neuroprotection for ischemic stroke clinically have failed [15].

The causes of the failure must be diverse. The effects of drugs may differ between animals and patients as a consequence of the differences in body sizes, circulatory systems, or brain physiologies. Or there may be a difference between the artificial animal ischemia model and human ischemia due to natural causes. Also, it is quite possible that pharmacological target molecules or events are inappropriately chosen in the failure cases. In this case, we have to seek out new drug targets that are different from those that were not successful.

CsA has a unique feature in that it targets mitochondrial dysfunction. Its action on MPT is gaining attention from many researchers. Given such circumstances, a new phase II clinical trial, “CsAStroke”, has begun, with the aim of evaluating CsA’s effect on the volume of cerebral infarction after intravenous thrombolysis [82]. CsA has been shown to exert a neuroprotective effect not only in ischemia but also in PD [135,182,183], AD [184], and neurons under oxidative stress [185]. CsA is known to enhance the regrowth of damaged peripheral and central neurons [186] and to increase muscular force generation in Duchenne muscular dystrophy [187,188].

Our findings revealed that, in rat brain, CsA appears to regulate the expression of many genes related to cell survival and regeneration. If we specify the transcriptional factor(s) responsible for this regulation, we should be able to mimic this transcriptional regulation without using CsA. Also, the characterization of this “survival pathway” may lead us to identify a novel system similar to neurotrophic factor signaling or an anti-apoptotic pathway. Consequently, CsA effects on transcriptional regulation and characterization of the genes discussed in our study will facilitate the development of new conceptual and pharmacological tools that target the activation of a novel neuroprotective mechanism based on the function of (still unidentified) transcriptional factor(s), such as PHB1 or similar molecules. On the other hand, we still do not know which transcriptional factor or factors is or are responsible for this CsA effect, nor have we tested known factors, such as calcineurin. We have not yet performed cDNA subtraction using FK506 to test the effect of calcineurin inhibition. Further investigations are required to figure out the mechanism underlying CsA’s effect on transcriptional regulation.

## Abbreviations

AD: Alzheimer’s disease
ALS: amyotrophic lateral sclerosis
ANT: adenosine nucleotide translocase
BDNF: brain-derived neurotrophic factor
CaM: calmodulin
CCP: classical complement pathway
CsA: Cyclosporin A
CypD: cyclophilin-D
G-CSF: granulocyte-colony stimulating factor
HD: Huntington’s disease
HETE: hydroxyeicosatetraenoic acid
HIF-1: hypoxia Inducible factor-1
IL-1: interleukin-1
IL-2: interleukin-2
MCAO: middle cerebral artery occlusion
MDH: malate dehydrogenase
MPT: mitochondrial membrane permeability transition
MS: multiple sclerosis
NF-AT: nuclear factor of activated T-cells
NMDA: *N*-methyl-D-aspartate
PARP: poly ADP-ribose polymerase
PD: Parkinson’s disease
PGK-1: phosphoglycerate kinase 1
SAH: subarachnoid hemorrhage
SCI: spinal cord injury
STAT3: signal transducers and activator of transcription 3
TBI: traumatic brain injury
VDAC: voltage dependent anion channel
VDC: voltage-dependent calcium channel
